# Does Inside Equal Outside? Relations Between Older Adults' Implicit and Explicit Aging Attitudes and Self-Esteem

**DOI:** 10.3389/fpsyg.2018.02313

**Published:** 2018-11-22

**Authors:** Jing Chen, Kangjia Zheng, Weihai Xia, Qi Wang, Zongqing Liao, Yutong Zheng

**Affiliations:** ^1^Research Center of Psychological Development and Application, Sichuan Normal University, Chengdu, China; ^2^School of Teacher Education and Psychology, Sichuan Normal University, Chengdu, China

**Keywords:** implicit attitude toward one's own aging, explicit attitude toward one's own aging, implicit self-esteem, explicit self-esteem, older adults

## Abstract

Attitudes toward one's own aging and self-esteem are crucial variables in predicting older adults' physical and mental health and can significantly affect their will to live, cognitive judgement and acceptance of medical treatment. However, little is known about the relation between the implicit attitude toward one's own aging and implicit self-esteem. This research explored consistencies between implicit and explicit attitudes toward one's own aging and between implicit and explicit self-esteem and explored their relations in 70 older adults aged 60–91 years old using the word and picture versions of the Implicit Association Test and standardized scales. The results showed that (a) the explicit and implicit attitudes toward one's own aging represented independent structures, and the implicit and explicit self-esteem also represented independent structures; (b) subjects generally showed positive explicit attitudes toward their own aging and negative implicit attitudes toward their own aging while also showing high explicit self-esteem and relatively low implicit self-esteem; (c) subjects' implicit attitudes toward their own aging and implicit self-esteem were positively correlated, and explicit attitudes toward their own aging and explicit self-esteem were also positively correlated. The more positive the subjects' explicit attitudes toward their own aging, the higher their explicit self-esteem levels were. The more negative their implicit attitudes toward their own aging, the higher their implicit self-esteem levels were. We concluded that older adults' explicit and implicit attitudes toward their own aging and self-esteem are independent structures; older adults' explicit and implicit attitudes toward their own aging have predictive effects on their explicit and implicit self-esteem in different directions, respectively.

## Introduction

With the advent of the twenty-first century, many countries have entered a stage of rapid population aging accompanied by serious population aging pressure. Challenged by this problem, all states must take positive measures to promote the maintenance and development of the independence of older adults' individual functions and improve their quality of life. One of the most important factors affecting older adults' quality of life is their subjective perception of their own aging. Therefore, to move toward the above goal, first, all related states must help older adults form positive attitudes about their own aging.

Since the concept of attitude toward aging embraces two layers of meaning, beliefs and assessments, attitude toward aging is divided into different categories based on these two areas. First, in terms of beliefs, attitude toward aging is also known as the aging stereotype, which reveals an individual's mental representation of the characteristics and behaviors of older adults (Kornadt et al., 2014). Second, regarding assessments, attitudes toward aging are classified into two categories: one's general attitude toward aging and one's attitude toward one's own aging. The former is the attitudes toward older adults held by individuals of different ages (the broadly defined attitude toward aging) whereas the latter indicates older adults' own attitudes toward themselves (the narrowly defined attitude toward aging; Laidlaw et al., 2007; Cheng et al., 2009). According to the classification and concept definitions introduced above, the subjects of the narrowly defined attitude toward aging are only older adults. The narrowly defined attitude toward aging refers to older adults' experience with aging, that is, their understanding and expectations of their own aging process and their current or future life in old age. This specific attitude can be used to predict their mental health level (Levy et al., 2002) and exerts enormous influence on older adults' physical and mental states, cognitive and physical performance (Hummert, 2003; Levy and Leifheitlimson, 2009).

The attitude toward one's own aging has positive (older adults are wise and mature) and negative aspects (older adults are weak and decrepit; Laidlaw et al., 2007). The positive effects on older adults of a positive attitude toward their own aging have been demonstrated in numerous studies. A positive implicit attitude improves their memory performance and memory self-efficacy (Levy, 1996) and a positive explicit attitude is positively correlated with older adults' better physical and emotional functioning, greater resilience, and less depression (Levy et al., 2002; Kavirajan et al., 2011). A study with Chinese older subjects has showed that implicit attitudes toward aging are positively and significantly correlated with the numerical working memory span, that is, the more positive the implicit aging attitudes, the higher the performance of span of numerical working memory (Yin et al., 2014). A negative attitude toward their own aging of the aged is termed “older adults' ageism” and includes the ideas that older adults perceive themselves as becoming weak, losing functions and social status, and becoming a burden on society (Kruse and Schmitt, 2006). This negative attitude is derived from both traditional concepts and the stagnation and despair elicited by physical and mental degeneration that can impede older adults' continuing development in two areas. On the one hand, this negative attitude (both explicit and implicit) can have negative effects on older adults' cognitive performance (Levy, 1996, 2003b; Levy et al., 2002; Chasteen et al., 2005; Lineweaver et al., 2009). For example, the activation of a negative implicit attitude toward their own aging negatively influences older adults' self-concept and their cognitive and physical performance (Levy, 1996, 2009). In addition, such an attitude (both explicit and implicit) reduces older adults' levels of subjective well-being and mental health (Levy et al., 2002; Levy, 2003a; Coudin and Alexopoulos, 2010; Scott et al., 2011). More importantly, older adults' attitude toward their own aging can directly affect their will to live. When faced with a hypothetical terminal illness, the older subjects who had negative implicit attitudes toward their own aging tended to be less willing to accept medical interventions than those who had positive implicit attitudes toward their own aging (Levy et al., 2000). In addition, the effect of a negative implicit attitude toward their own aging on older adults' will to live is persistent (Marques et al., 2014). Consequently, overcoming older adults' negative attitude toward their own aging has great meaning in maintaining and promoting their social adaptation and mental health. Given the above, the first purpose of this study was to investigate older adults' attitudes toward their own aging from a Chinese perspective.

Based on the literature analysis, some limitations can be found in related research areas. First and foremost, most studies used questionnaires (e.g., Cherry and Palmore, 2008) or interviews (e.g., Romo et al., 2013) to investigate older adults' explicit attitudes toward their own aging and paid scant attention to the Implicit Association Test (IAT) to investigate their implicit attitudes toward their own aging. Nevertheless, much research has demonstrated that the activation of stereotypes will appear on both the explicit and the implicit levels (Dijksterhuis et al., 2000; Wheeler and Petty, 2001; Jonas and Sassenberg, 2006). In addition, compared with the questionnaire survey method, which can only measure individual subjective attitudes and may be affected by social desirability, the use of the IAT to measure individual implicit attitudes has been shown to avoid the information distortion caused by self-presentation so that subjects show their true attitudes (Levy, 2003a; Lin et al., 2010; Calanchini and Sherman, 2013). In particular, some research has demonstrated that implicit attitudes toward one's own aging were more negative than explicit attitudes and created more negative influences (Nosek et al., 2002; Hess et al., 2004; Meisner, 2012). For example, the two types of attitudes toward one's own aging can influence older adults' memory performance; however, the implicit attitude has been shown to exert more significant effects than the explicit attitude (Hess et al., 2004). Another study has provided evidence that those implicit and explicit attitudes toward aging apparently represent independent structures (Kornadt et al., 2014). A study with a Chinese sample determined that when the education level is controlled, the older a subject is, the more negative his/her implicit attitudes toward aging are, and the degrees of implicit aging stereotypes of older subjects are significantly higher than those of younger subjects (Zhou, 2007). It follows that older adults' implicit and explicit attitudes toward their own aging may well-represent independent structures. It is thus obvious that the use of IAT and standardized scales or inventories to explore older adults' implicit and explicit attitudes toward their own aging and their relationship is a feasible approach to resolving the above limitation and has great research significance. Second, with regard to the study subjects, although several studies have been conducted to investigate young adults' general attitude toward aging, little attention has been directed toward older adults' attitudes toward their own aging. Third, in terms of research content, although much attention has been paid to depicting the characteristics of older adults' attitudes toward their own aging, little has been done to explore the consistency of attitudes' inner psychological components. Based on the above analysis, the second purpose of this research was to investigate older adults' implicit and explicit attitudes toward their own aging and the consistency of those attitudes.

Self-esteem is a judgement of value demonstrated by attitudes about an individual's self and may be closely related to self-worth (Beebe et al., 2011). It reflects the extent to which individuals perceive themselves to be successful, competent and valuable (Kernis and Goldman, 2005), and it is one of significant factors in individual social adaptation (van Tuijl et al., 2014). Individuals with high self-esteem tend to perceive themselves with respect and approval and, significantly, as beneficial (Arslan et al., 2010). Such people thus approach problems in a positive manner (D'zurilla et al., 2003). Conversely, people with low self-esteem generally lack confidence and tend to make negative assessments of their own competence (Wrench et al., 2008). According to the Terror Management Theory (Greenberg et al., 1986), self-esteem has the effect of relieving negative emotions (anxiety of mortality). The purpose and function of self-esteem are to reduce and alleviate anxiety; thus, it becomes a determinant for the mental health of older adults (Greenberg et al., 1992). For example, the results of a meta-analysis of 95 longitudinal studies showed that low self-esteem could predict levels of both depression and anxiety (Sowislo and Orth, 2013). Individuals with low explicit self-esteem tend to have high social anxiety and show defensive cognition and affective responses in interpersonal interactions (van Tuijl et al., 2014). In conclusion, older adults' self-esteem plays an important role in their mental health and social adaptation.

Much research has presented evidence of a significant decline in self-esteem levels as individuals move into old age (Robins et al., 2002; McMullin and Cairney, 2004; Orth et al., 2010; Wagner et al., 2013). Is this decline in self-esteem related to older adults' attitudes toward their own aging? This is precisely the interesting question addressed by the current research. In relevant research fields, little research has identified a relation between older adults' self-efficacy and attitudes toward their own aging and their behaviors. Research results indicated that when older adults had positive attitudes toward their own aging or possessed a low level of negative implicit aging stereotypes, they tended to think that they could manage all their affairs based on their abundant experience, which promotes their self-efficiency and leads to better cognitive performance (Levy, 1996; Hess et al., 2003). Those older adults strongly believe themselves to be active, vital and healthy members of society, and they have a tendency to take better care of themselves by maintaining good eating habits and exercise patterns (Del Villar, 2014). Clearly, the attitude toward one's own aging and the self-esteem of the elderly jointly play an important role in older adults' physical and mental health. To date, however, to our knowledge, few studies have directly investigated the relations between older adults' self-esteem and their attitudes toward their own aging. One study used the questionnaire method to determine that older adults with positive explicit attitudes toward their own aging tended to display high explicit self-esteem (Del Villar, 2014). Carmencita (2014) proved that self-esteem could be predicted by the elders' attitudes about aging. The other longitudinal study indicated that attitude toward one's own aging affect older adults' self-concept (Levy et al., 2002): they may make negative self-evaluations and behaviors because of automatically accepting negative aging stereotypes (Levy, 2003a, 2009). Thus, it can be inferred that older adults' attitudes toward their own aging may predict their level of self-esteem. The third purpose of the current research, then, was to investigate the relation between older adults' attitudes toward their own aging and self-esteem and whether their attitudes toward their own aging influenced their self-esteem level.

Previous studies have also demonstrated that implicit and explicit self-esteem are different types of self-evaluation: the former refers to one's conscious evaluation of the self; the latter is one's automatic affective or evaluative associations with the self (Bosson et al., 2000; Greenwald and Farnham, 2000). Implicit self-esteem has also become one of the crucial psychological indicators to measure an individual's level of self-esteem (Greenwald and Banaji, 1995). However, relevant research on self-esteem with samples of old Chinese adults is rare; few studies have focused on older adults' explicit self-esteem rather than their implicit self-esteem. For example, one study determined that older adults' explicit self-esteem was comparable to that of middle-aged adults and was influenced by gender and education level (Li et al., 2016). Another study indicated that older adults had relatively high explicit self-esteem (Shen et al., 2003; Han, 2017). Therefore, the self-esteem that we investigated in the current research included both the implicit and explicit self-esteem of older adults.

As discussed above, implicit attitudes have attracted much attention in research domains of both attitudes toward aging and self-esteem. The Implicit Association Test (IAT) is used most often to measure implicit attitudes. Nevertheless, most studies have used the Word IAT designed by Greenwald et al. (1998) (e.g., Greenwald and Farnham, 2000; Cai, 2003a,b; Lin et al., 2010; Kornadt et al., 2014). Only a few studies have used the Picture IAT (e.g., Foroni and Bel-Bahar (2010); Hass and Lim (2017) adopted the Picture IAT to investigate age-related implicit attitudes but only recruited college students to investigate their general attitudes toward aging. Compared with the Word IAT, the use of the Picture IAT for measuring implicit attitudes presents stimuli with higher levels of representation; therefore, subjects experienced more real stimuli and thus displayed stronger tendencies (Foroni and Bel-Bahar, 2010). Moreover, because older adults may experience cognition aging and memory degradation, a large amount of word materials might elicit a high cognitive load, which renders it difficult for them to recognize and judge, and then leads to false negative errors. For this reason, to reduce older subjects' cognitive load and enhance ecological validity, this study, although using the classic Word IAT, developed the Picture IAT for elderly Chinese subjects to investigate older subjects' implicit attitudes toward their own aging.

Considering the above analyses and research purposes, the current research includes two studies established to study the consistency between older adults' implicit and explicit attitudes toward their own aging, the consistency between their implicit and explicit self-esteem, and to study the relation between their attitudes toward their own aging and self-esteem using the IAT paradigm and standardized measurement tools. Study 1 investigated the implicit and explicit attitudes toward their own aging of older subjects and their consistency with Attitudes toward One's Own Aging Subscale and Word and Picture IATs. In Study 2, we employed the Self-esteem Inventory and IAT to investigate the implicit and explicit self-esteem of the same subjects as in Study 1 and their consistency. Finally, we conducted an integrative analysis of the two studies' data to investigate the relationship between implicit-explicit attitudes toward one's own aging and implicit-explicit self-esteem in older adults. Based on the literature review and our analysis, five main hypotheses were proposed: (a) older adults' explicit and implicit attitudes toward their own aging are two independent structures, (b) subjects of this study would have positive explicit and negative implicit attitudes toward their own aging, (c) older adults' implicit and explicit self-esteem would demonstrate two independent structures, (d) subjects in our sample would have high explicit self-esteem and relatively low implicit self-esteem, (e) older adults' explicit attitude toward one's own aging positively predicts explicit self-esteem and their implicit attitude toward one's own aging score (*D*) positively predicts the implicit self-esteem score.

## Study 1

### Methods

#### Subjects

Seventy-four subjects aged 60–91 years old were recruited voluntarily from a nursing home, two communities, an old people's living center, and a college for the aged in Chengdu, China. The inclusion criteria for all subjects were (a) right-handedness, (b) normal sight (included the subjects with corrected-to-normal sight), (c) abilities to respond on the keyboard with instruction, (d) no known physiological illness that may affect the study, and (e) not having been engaged in similar studies previously. Four subjects failed to complete the experiment or showed high error rates (above 20%). Hence, their data were excluded from the final data analysis. Consequently, we collected valid data from seventy subjects (*M*_*Age*_ = 72.77, *SD* = 9.504; 30 males), which accounts for 94.59% of the total sample. Written informed consent was signed by all participants. The subjects' information is listed in Table 1.

**Table 1:** Subjects' characteristics (*N* = 70).

**Demography variable**		**Age (*M ± SD*)**	**Proportion (%)**
Gender	Male (*n* = 30)	73.50 ± 9.74	42.86
	Female (*n* = 40)	72.00 ± 9.40	57.14
Age	Under 74 (*n* = 38)	65.21 ± 4.71	54.29
	Above 74 (*n* = 32)	81.75 ± 4.61	45.71
Education level	Junior Middle School or Below (*n* = 21)	73.29 ± 9.64	30.00
	High School (*n* = 21)	70.19 ± 8.99	30.00
	Bachelor Degree or Above (*n* = 28)	74.32 ± 9.71	40.00

#### Design

The IAT was employed to investigate subjects' implicit attitudes toward their own aging. Subjects were required to judge the classification of the concept words and the target words. We then examined whether a significant difference in response latency would emerge between when subjects reacted to the combination of age-related concepts and negative attributes and the combination of age-related concepts and positive attributes. The standardized scale was also used to measure subjects' explicit attitudes toward their own aging. This study was conducted in accordance with the recommendations of “The Research Ethical Principles for Human Subjects, the Institution Research Ethics Board of the University” with written informed consent from all subjects. All subjects gave written informed consent in accordance with the Declaration of Helsinki. The protocol was approved by the university.

### Materials

#### IAT tasks

**Figure 1:**
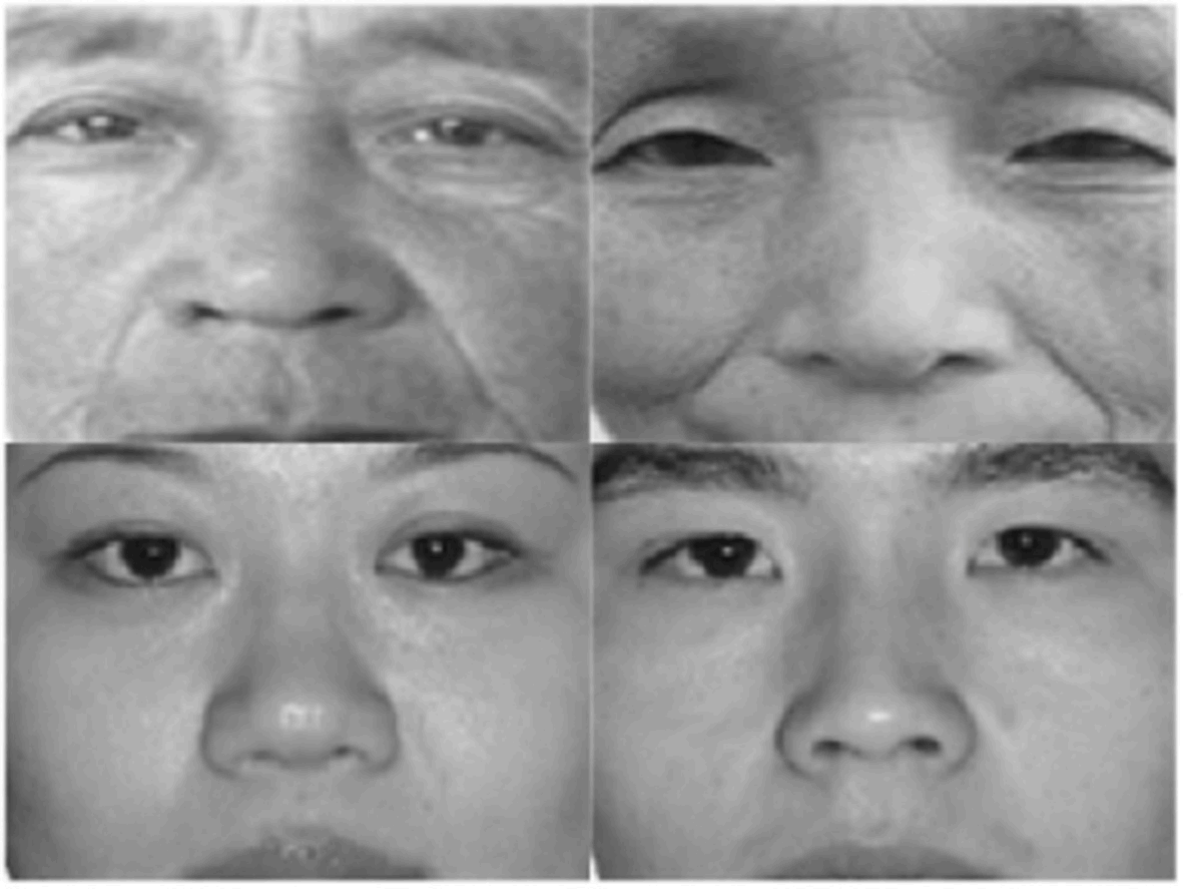
Pictures with neutral facial expressions were presented to subjects. Pictures were created following the process of a classic IAT experiment (Hummert et al., 2002). 167 pictures with neutral facial expressions of older adults were taken by SLR cameras by us. Then, 15 older adults and 15 college students were invited to rate these pictures on a 7-point Likert-type scale based on emotional status and the awakening level of the expressions. We then calculated the means and standard deviations and used six pictures with scores within one standard deviation as experimental materials. The pictures of young people were selected from the Chinese Affective Picture System (Bai et al., 2005). An additional processing was performed on the pictures, including removing the background and saving the face from the eyebrows to the upper lip. The pictures were then converted to black and white photos at a size of 130 × 97 pixels.

Two IAT tasks were designed to measure older adults' implicit attitudes toward their own aging. Consistent with the research purposes and the classic Implicit Association Test (IAT), we devised two versions of IAT tasks: Word IAT in which the stimuli were all words and Picture IAT, which comprised pictures as conceptual stimuli and words as target stimuli. (a) In Word IAT, words comprised concept and target words. According to the classical paradigm of Greenwald et al. (1998), we selected six “old” concept and six “young” concept words. The “old” concept words included *old, older, aged, old people, the aged*, and *old age*. The “young” concept words included *youth, young, juvenile, juvenescence, young people*, and *younger*. The attitude toward aging involves measuring one's experience and evaluation of old age, which shares much in common with the implicit self-esteem study that measures one's positive and negative experiences and evaluations of the self and others. Therefore, all the target words were chosen from the classic IAT study (Greenwald et al., 1998; Greenwald and Farnham, 2000; Cai, 2003a,b), including 10 positive words (*health, joy, smart, bright, success, noble, strong, worthy, competent*, and *valued*) and 10 negative words (*weak, awkward, filth, ugly, agony, stupid, failure, ashamed, rotten*, and *useless*). (b) The Picture IAT used pictures of neutral facial expressions of old adults and young adults as stimuli. The pictures of old adults were developed according to the procedure of a picture IAT experiment (Hummert et al., 2002). The production occurred in several steps. First, we took 167 pictures with neutral facial expressions of older adults with SLR cameras. Then, we invited 15 old adults and 15 college students to rate these pictures on a 7-point Likert-type scale based on emotional status and arousal level. The emotional status refers to the positive or the negative emotion that the raters think the pictures represent. The score ranges from 1 to 7(from negative to positive). The arousal level indicates the excited level that the raters feel when see these pictures, the score ranges from 1 to 7 (from calm to excited). We then calculated the means and standard deviations and used six pictures with scores within one standard deviation as experimental materials (emotional status: *M* = 3.33, *SD* = 0.17; arousal level: *M* = 2.13, *SD* = 0.30). The pictures of young people were selected from the Chinese Affective Picture System (Bai et al., 2005). Finally, additional processing was performed on the pictures, including removing the background and saving the face from the eyebrows to the upper lip. The pictures were then converted to black and white photos in a size of 130 × 97 pixels (see Figure 1). The Chinese-modified professional IAT software Inquisit 2.0 (Geng, 2009) was used to design the program used in the experiment; the program could automatically record response latency and the accuracy of the subjects.

#### Attitudes toward one's own aging subscale

We used the Chinese version of the Attitudes Toward Own Aging Subscale of the Philadelphia Geriatric Center Morale Scale (Lawton, 1975) to measure the participants' explicit personal aging attitudes (Yao et al., 1995) (see Appendix). The PGC comprises 23 forced-choice items and 3 factors, including agitation, dissatisfaction and attitudes toward one's own aging; and the test-retest reliability is 0.804 (Yao et al., 1995). To measure older adults' attitudes toward their own aging, the “attitudes toward one's own aging” factor was used; its Chinese version includes 7 items, and the scores range from 0 to 7. A higher score indicates a more positive attitude toward one's own aging, referring to individuals who think the positive effects of aging outweigh the negative effects (Yao et al., 1995). The Cronbach's α of the scale in this study was 0.636. Sample items included “things get worse as one ages,” “I have as much pep as last year,” and “As one ages, he becomes less useful.”

### Procedure

The study was conducted individually. After the instructions were explained, subjects began to take the test. Their eyes were ~45 cm away from the computer screen. All stimuli were presented in the screen center. Both the Word IAT and Picture IAT contained seven blocks (see Table 2; Greenwald et al., 2003). Each subject was required to complete both Word and Picture IATs, and the accuracy and the response latency were recorded automatically by the computer program. To control the sequence effect, the compatible and non-compatible tasks were presented to half of the subjects in the opposite order. After finishing the IAT, subjects were given 10 min to rest, and then they completed the Attitudes toward One's Own Aging Subscale.

**Table 2:** Examples used in the implicit association test.

**Order**	**Block**	**Stimuli words**	**Response key**
1	General practice block: initial target-concept discrimination	Young	E
		Old	I
2	General practice block: associated attribute discrimination	Positive	E
		Negative	I
3	Practice block: initial combined block 1	Young and Positive	E
		Old and Negative	I
4	Critical block: initial combined block 2	Young and Positive	E
		Old and Negative	I
5	General practice block: reversed target-concept discrimination	Old	E
		Young	I
6	Practice block: reversed combined block 3	Old and Positive	E
		Young and Negative	I
7	Critical block: reversed combined block 4	Old and Positive	E
		Young and Negative	I

### Data analysis

According to the research of Greenwald et al. (2003), the data of IAT were analyzed as follows. The data of subjects who did not complete all experiments and whose average accuracy was lower than 80% were removed. The data from subjects for whom more than 10% of the trials had a latency of < 300 ms or more than 10,000 ms were excluded, and each error reaction was replaced with the block mean time plus 600 ms. Accordingly, the total standard deviation of the two practice blocks (Nos. 3 and 6) and two critical blocks (Nos. 4 and 7) was calculated separately. D1 was obtained by taking the difference in mean response latency between the two practice block conditions (Nos. 3 and 6) and scaling it by the participant's average latency standard deviation for both blocks (Nos. 3 and 6). Likewise, D2 was calculated by taking the difference in mean response latency between the two critical blocks (Nos. 4 and 7) and divided by the standard deviation of both blocks (Nos. 4 and 7). Finally, D was calculated by averaging D1 and D2. Higher values of D indicate a stronger tendency of the implicit attitude toward own aging. The internal consistency was calculated by excluding the data of the first two practice blocks and the first two critical blocks, and the remaining data were then used to calculate α coefficient. Data from the Attitudes toward Own Aging Subscale were processed according to its scoring method.

### Results

Subjects' performance on the compatible and non-compatible blocks is presented in Table 3. Significant differences were observed between the scores of the compatible and non-compatible blocks in both Word and Picture IATs (Word IAT, *t* = −2.211, *df* = 69, *p* = 0.030 < 0.05, *d* = 0.374, *G power* = 0.707; Picture IAT, *t* = −2.306, *df* = 69, *p* = 0.024 < 0.05, *d* = 0.390, *G power* = 0.738). Moreover, subjects' response latency was shorter in the conditions of “young” concept words + positive words and “old” concept words + negative words (the compatible combination) whereas their response latency was longer in the conditions of “young” concept words + negative words and “old” concept words + positive words (the non-compatible combination). This suggests that the aging stereotype (negative implicit attitude toward one's own aging) is prevalent among these subjects, and they tend to link the “old” concept with negative attributes.

**Table 3:** Response latency of the compatible and non-compatible blocks (*N* = 70).

**Block type**	**Response latency of the compatible block (*****s*****)**		**Response latency of the non-compatible block (*****s*****)**		***D***		***t***	**α**
	***M***	***SD***	***M***	***SD***	***M***	***SD***	
Word IAT	1434.44	661.92	1690.99	811.28	0.30	0.75	−2.21^*^	0.85
Picture IAT	1388.03	521.32	1581.75	754.08	0.26	0.52	−2.306^*^	0.91

* *p < 0.05. The compatible blocks are categories of Young concept words and positive words, the non-compatible blocks are categories of Young concept words and negative words*.

The mean score of the explicit attitude toward one's own aging in our sample was 5.24 ± 1.619, which indicates that the subjects generally showed positive explicit attitudes toward their own aging. We then used demographic variables (gender, age, education level) as independent variables and the explicit and implicit attitudes toward one's own aging as dependent variables for the analysis of variance (ANOVA). The results are as follows: (a) For the explicit attitude toward one's own aging score, the main effects of three demographic variables were insignificant. Gender, *F*_(1, 69)_ = 2.969, *p* = *0.0*90 > 0.05, η^2^ = 0.049, *G power* = 0.174; age, *F*_(1, 69)_ = 0.159, *p* = 0.692 > 0.05, η^2^ = 0.003, *G power* = 0.055; educational level, *F*_(2, 69)_ = 0.736, *p* = 0.484 > 0.05, η^2^ = 0.025, *G power* = 0.104. And the interaction effects of them were insignificant. Gender and age, *F*_(1, 69)_ = 2.752, *p* = *0.1*03 > 0.05, η^2^ = 0.045, *G power* = 0.161; gender and educational level, *F*_(2, 69)_ = 0.428, *p* = 0.654 > 0.05, η^2^ = 0.015, *G power* = 0.080; age and educational level, *F*_(2, 69)_ = 1.555, *p* = 0.220 > 0.05, η^2^ = 0.051, *G power* = 0.1*80*. (b) Regarding the score of implicit attitudes toward one's own aging (Word IAT), the main effect of gender was significant, *F*_(1, 69)_ = 6.331, *p* = 0.015 < 0.05, η^2^ = 0.098, *G power* = 0.361; but the main effects of age and educational level were both insignificant, *F*_(1, 69)_ = 0.215, *p* = 0.644 > 0.05, η^2^ = 0.004, *G power* = 0.057; *F*_(2, 69)_ = 0.741, *p* = 0.481 > 0.05, η^2^ = 0.025, *G power* = 0.104. Additionally, the interaction effects of the three demographic variables were insignificant. Gender and age, *F*_(1, 69)_ = 0.092, *p* = 0.763 > 0.05, η^2^ = 0.002, *G power* = 0.054; gender and educational level, *F*_(2, 69)_ = 0.705, *p* = 0.498 > 0.05, η^2^ = *0.0*24, *G power* = 0.101; age and educational level, *F*_(2, 69)_ = 0.188, *p* = 0.829 > 0.05, η ^2^ = 0.006, *G power* = 0.061. (c) Regarding the scores of implicit attitudes toward one's own aging (Picture IAT), the main effects of three demographic variables were insignificant. Gender, *F*_(1, 69)_ = 1.760, *p* = 0.190 > 0.05, η^2^ = 0.029, *G power* = 0.114; age, *F*_(1, 69)_ = 3.719, *p* = 0.059 > 0.05, η^2^ = 0.060, *G power* = 0.211; educational level, *F*_(2, 69)_ = 1.772, *p* = 0.179 > 0.05, η^2^ = 0.058, *G power* = 0.204. And the interaction effects of them were insignificant. Gender and age, *F*_(1, 69)_ = 0.870, *p* = 0.355 > 0.05, η^2^ = 0.015, *G power* = 0.080; gender and educational level, *F*_(2, 69)_ = 1.023, *p* = 0.366 > 0.05, η^2^ = 0.034, *G power* = 0.128; age and educational level, *F*_(2, 69)_ = 0.279, *p* = 0.758 > 0.05, η^2^ = *0.0*10, *G power* = 0.069.

To test the consistency between subjects' implicit and explicit attitudes toward their own aging, a Pearson correlation analysis was performed. The analysis showed a significant correlation between the scores of the Word and Picture IAT tasks, *r* = 0.273, *p* = 0.022 < 0.05, *G power* = 0.760, but no significant correlation was identified between the scores of Word and Picture IAT tasks and explicit attitudes toward one's own aging, *r*_*word*_ = −0.157, *p* = 0.195 > 0.05, *G power* = 0.371; *r*_*picture*_ = −0.022, *p* = 0.856 > 0.05, *G power* = 0.072. These results demonstrate a consistency between the Word and Picture IATs and an inconsistency between the implicit and explicit attitudes toward the subjects' own aging.

To test the validity of the Picture IAT developed for this study, we conducted a *t-*test on the scores of both the Word and Picture IATs, but no significant difference was identified (*t* = 0.448, *df* = 69, *p* = 0.655 > 0.05, *d* = 0.0758, *G power* = 0.115). This demonstrates that the measuring results of the Picture IAT were basically consistent with the results of the Word IAT: the Word and Picture IAT tasks were clearly consistent in the current study.

### Discussion

The results of Study 1 support the hypotheses proposed by the current research. First, the subjects generally showed positive attitudes toward their own aging, but they tended to link the “young” concept with positive attributes and link the “old” concept with negative attributes, which reveals their implicit negative attitudes toward their own aging, and is consistent with previous research conducted on subjects with same cultural background (e.g., Zhou, 2007). Moreover, the conclusion that the explicit and implicit attitudes toward one's own aging are independent and fall into different structures can be deduced from the result that there is no significant correlation between them in the same subjects, which is also consistent with previous research (e.g., Kornadt et al., 2014). Third, the picture stimuli for the Picture IAT were self-created while the word stimuli for the Word IAT were chosen from previous IAT studies. The results showed no significant difference between the scores of the two IAT tasks, which provides support for the validity of the Picture IAT. The consistency between the Word and Picture IAT tasks indicates that both Word and Picture IAT tasks could be used together to effectively measure implicit attitudes toward one's own aging.

It is worth noting that a previous study with a sample of 90 old Chinese adults reported that the average score of explicit attitude toward one's own aging was 4.9889 ± 1.59 (Yao et al., 1995), and the average score of the current study (5.24 ± 1.619) was nearly the same. In addition, a study on the explicit attitude toward one's own aging with 203 old Chinese adults reported that the mean score of explicit attitude toward one's own aging in the 108 subjects aged 60–70 years old was 3.83 ± 1.49, and in the 95 subjects aged above 71 years old, the mean score was 3.35 ± 1.41. Such data indicate that the older subjects in our study demonstrated more positive explicit attitudes toward their own aging than old subjects with the same cultural background in previous research. This may be connected with the fact that more than two-thirds of the subjects in our sample had a relatively high education level.

## Study 2

### Methods

#### Subjects

The same group of subjects were used as in Study 1.

#### Design

The IAT was employed to measure implicit self-esteem. Subjects were required to make judgements on the classification of the concept words and the target words. We then examined whether a difference occurred between when subjects reacted to the combination of self-concept and positive attributes (compatible) and the combination of self-concept and negative attributes (non-compatible). A standardized inventory was used to measure subjects' explicit self-esteem.

### Materials

#### IAT task

An IAT task was designed with reference to the classic IAT paradigm (Greenwald et al., 1998) to measure implicit self-esteem. The stimuli included concept words and target words. Consistent with the self-esteem IAT (Greenwald et al., 1998), slight replacements were made to the target words. The concept words contained “self” concept words (*I, my, me, mine, self, myself* ) and “other” concept words (*he, she, they, them, their, other*). The target words that contained positive and negative words were the same as in Study 1.

#### The self-esteem inventory

Given that the Self-esteem Scale (SES) (Rosenberg, 1965) is more applicable to individuals below high school age (Wang et al., 1999), the eighth item was originally reverse-scored but is often understood to be positive-scored in the Chinese culture (Wang and Yang, 2007). Due to these limitations of the SES, we used the Chinese version of the Self-esteem Inventory (SEI) developed by Coopersmith (1967) to measure explicit self-esteem (Wang et al., 1999) (see Appendix). The inventory has been revised and demonstrated to be fit for measuring the self-esteem of adults (Ryden, 1978). Its split-half reliability in existing studies with Chinese subjects has been shown to be 0.7537 and 0.73. The Cronbach's α was 0.76 (Zhang, 1997; Cai, 2003b). The inventory comprises 58 items, eight of which are reverse-scored. Each item requires subjects to answer with “like me” or “unlike me.” Each response indicating a positive attitude toward one's self was marked as 1, and negative responses were scored as 0. The full score ranged from 0 to 58. A higher total score indicated higher self-esteem. The Cronbach's α coefficient was 0.88 (Wang et al., 1999). In this study, the Cronbach's α was 0.78. Sample items included “I spend a lot of time daydreaming”, “I am pretty sure of myself,” and “I often wish I were someone else.”

### Procedure

The two studies were conducted at an interval of 1 week. The procedure of finishing the human-computer interaction experiment and completing the inventory was consistent with Study 1. The sequencing of the concept and target words in the self-esteem IAT was the same as in Study 1. The difference was that the “old” and “young” concept words were replaced by the concept words of “self” and “other.” The target words were the same as in Study 1.

### Data analysis

The data processing methods of IAT were consistent with Study 1. Data from the SEI were processed according to its scoring method.

### Results

The subjects' performance in the compatible and non-compatible blocks is presented in Table 4. A significant difference was demonstrated between the compatible and the non-compatible blocks (*t* = −4.090, *df* = 69, *p* < 0.0001, *d* = 0.691, *G power* = 0.992): subjects responded faster when they put the “self” concept words in the same category as positive words and put the “other” concept words in the same category as negative words (the compatible combination) whereas they responded more slowly when they put the “self” concept words in the same category as negative words and put the “other” concept words in the same category as positive words (the non-compatible combination). This demonstrates a significant and notable self-esteem effect among the subjects, who tended to link the “self” concept with positive attributes and link the “other” concept with negative attributes.

**Table 4:** Response latency in the compatible and non-compatible blocks (*N* = 70).

**Block type**	**Response latency of the compatible block (*****s*****)**		**Response latency of the non-compatible block (*****s*****)**		***D***		***t***	**α**
	***M***	***SD***	***M***	***SD***	***M***	***SD***	
IAT	1207.01	347.60	1388.18	448.26	0.22	0.45	−4.09^*^	0.85

* *p < 0.05. The compatible blocks are categories of self concept words and positive words, the non-compatible blocks are categories of other concept words and negative words*.

The mean explicit self-esteem score in this study was 41.21 ± 6.04. This high score indicated that the subjects showed high explicit self-esteem. Using gender, age, and education level as the independent variables and explicit and implicit self-esteem as dependent variables for ANOVA, the results are shown below. (a) Concerning the explicit self-esteem score, the main effects of three demographic variables were insignificant. Gender, *F*_(1, 69)_ = 2.738, *p* = 0.103 > 0.05, η^2^ = 0.045, *G power* = 0.161; age, *F*_(1, 69)_ = 0.476, *p* = 0.493 > 0.05, η^2^ = 0.008, *G power* = 0.065; educational level, *F*_(2, 69)_ = 1.876, *p* = 0.162 > 0.05, η^2^ = 0.061, *G power* = 0.215. And the interaction effects of them were insignificant too. Gender and age, *F*_(1, 69)_ = 0.792, *p* = 0.377 > 0.05, η^2^ = 0.013, *G power* = 0.075; gender and educational level, *F*_(2, 69)_ = 0.879, *p* = 0.421 > 0.05, η^2^ = 0.029, *G power* = 0.114; age and educational level, *F*_(2, 69)_ = 0.644, *p* = 0.529 > 0.05, η^2^ = 0.022, *G power* = 0.096. (b) Regarding the implicit self-esteem score, the main effect of gender was significant, *F*_(1, 69)_ = 9.324, *p* = 0.003 < 0.05, η^2^ = 0.138, *G power* = 0.534; men scored higher than women did. However, the main effects of age and educational level were insignificant. Age, *F*_(1, 69)_ = 0.128, *p* = 0.722 > 0.05, η^2^ = 0.002, *G power* = 0.054; educational level; *F*_(2, 69)_ = 0.837, *p* = 0.438 > 0.05, η^2^ = 0.028, *G power* = 0.111. Furthermore, the interaction effects of the three demographic variables were insignificant. Gender and age, *F*_(1, 69)_ = 1.717, *p* = 0.195 > 0.05, η^2^ = 0.029, *G power* = 0.114; gender and educational level, *F*_(2, 69)_ = 0.042, *p* = 0.959 > 0.05, η^2^ = 0.001, *G power* = 0.052; age and educational level, *F*_(2, 69)_ = 0.135, *p* = 0.874 > 0.05, η^2^ = 0.005, *G power* = 0.059.

To test the correlation of implicit and explicit self-esteem, a Pearson correlation analysis was performed. The results showed that there was no significant correlation (*r* = −0.011, *p* = 0.930 > 0.05, *G power* = 0.060), which indicates that implicit and explicit self-esteem are mutually independent.

### Discussion

The results of Study 2 support our hypotheses. The subjects showed high explicit self-esteem. This is not only consistent with domestic research results (e.g., Shen et al., 2003) but also provides evidence supporting the Theory of Adult Psychological Maturation (Gove et al., 1989). According to the maturational perspective, with increased age, individuals become more accepting of themselves and their situations, and their self-esteem tends to stabilize or even increase. Moreover, a study found that older adults' self-esteem has resilience; when faced with the loss of health, financial security or a career, they are able to adopt certain cognitive strategies to maintain their positive self-evaluation, such as social integration and downward comparison strategies (Collins and Smyer, 2005).

With regard to implicit self-esteem, subjects showed a typical implicit self-esteem effect: they tended to link the “self” concept to positive attributes and link the “other” concept to negative ones. The correlation between each subject's implicit and explicit self-esteem was insignificant, indicating that they are two independent structures. The above results are consistent with the results of previous research (e.g., Greenwald and Farnham, 2000). However, it is worth remembering that the effect size of implicit self-esteem in our study was lower than the effect size in relevant research with samples of Chinese or non-Chinese young adults (Greenwald and Farnham, 2000; e.g., Cai, 2003a,b) and lower than the effect size in non-Chinese old adults (e.g., Hummert et al., 2002). We suggest that this difference may derive from two factors. First, the above differences may arise from the differences between Eastern and Western cultures. Western culture emphasizes individualism and personal features, and self-evaluations of Westerners are accordingly high. Conversely, Eastern culture focuses more on collectivism and humility; therefore, Chinese people's self-evaluations are generally low. The differences may also stem from the age difference. A previous study (e.g., Wagner et al., 2013) identified a substantial self-esteem decline when individuals entered old age; the results of the current study may reflect that a decline of implicit self-esteem exists to some degree in older adults compared with young adults. According to theories of self-regulation, older adults are increasingly confronted with transitions or critical life events that are out of their control, particularly concerning declining health or social losses (e.g., bereavement). These challenges may simply become too frequent or too severe for them, rendering adjustment by self-regulation increasingly difficult. In addition, due to cognitive limitations, the capabilities of the self-regulation system may become more fragile with increased age. Consequently, the self-esteem of older adults shows a degree of general decline compared with that of young adults (Wagner et al., 2013).

## Analysis of the relation between attitude toward one's own aging and self-esteem

To further explore the relation between older adults' attitudes toward their own aging and self-esteem, the sum of the Word and Picture IAT scores were used as each subject's implicit attitudes toward their own aging score. The descriptive statistics of each variable in this study are presented in Table 5. We then conducted a Pearson correlation analysis of these variables (Table 6). Table 6 shows that there are significant and positive correlations between explicit self-esteem and explicit attitude toward one's own aging and between implicit attitude toward one's own aging and implicit self-esteem.

**Table 5:** The descriptive statistics of each measurement variable (*N* = 70).

	***Minimum***	***Maximum***	***M***	***SD***
Explicit attitude toward own aging	1	7	5.24	1.619
Implicit attitude toward own aging (total score)	−1.86	2.76	0.5544	1.017
Explicit self-esteem	28.00	50.00	41.2143	6.0406
Implicit self-esteem	−0.95	1.22	0.2188	0.45117

**Table 6:** Correlations between each measurement variable (*N* = 70).

	**Explicit attitude toward own aging**	**Implicit attitude toward own aging**	**Explicit self-esteem**	**Implicit self-esteem**
**Explicit attitude toward own aging**	1		
**Implicit attitude toward own aging**	−0.126	1	
**Explicit self-esteem**	0.258^*^	−0.141	1
**Implicit self-esteem**	−0.007	0.528^**^	−0.011	1

* *p < 0.05*,

** *p < 0.01*.

Based on the above analysis, controlling for gender, age, and education level, explicit and implicit attitudes toward one's own aging were entered as the predictors, and explicit and implicit self-esteem were taken as the outcome variables for a hierarchical regression analysis. The results are shown in Tables 7, 8.

**Table 7:** The regression analysis of the effect of explicit attitude toward own aging on explicit self-esteem.

	**Explicit self-esteem**			
	***ΔR^2^***	**β**	***t***	***G power***
Model 1	0.067			0.717
Explicit attitude toward own aging		0.258	2.205^*^
Model 2	0.065			0.705
Gender		0.172	1.442
Age		0.179	1.538
Education level		−0.097	−0.838
Explicit attitude toward own aging		0.237	1.982

* *p < 0.05*.

**Table 8:** The regression analysis of the effect of implicit attitude toward own aging on implicit self-esteems.

	**Implicit self-esteem**			
	***ΔR^2^***	**β**	***t***	***G power***
Model 1	0.268			0.999
Implicit attitude toward own aging		0.528	5.127^***^
Model 2	0.271			0.999
Gender		−0.185	−1.704
Age		0.013	0.125
Education level		−0.068	−0.637
Implicit attitude toward own aging		0.449	3.985^***^

*** *p < 0.001*.

According to Table 7, explicit attitude toward one's own aging can significantly predict and explain 6.7% of the variance of explicit self-esteem, and the β value of the explicit attitude toward one's own aging is positive, which indicates that the explicit attitude toward one's own aging positively predicts explicit self-esteem. Therefore, a more positive explicit attitude toward one's own aging indicates a higher explicit self-esteem. According to Table 8, implicit attitude toward one's own aging can significantly predict and explain 26.8% of the variance of implicit self-esteem; and the β value of the implicit attitude toward one's own aging in the model was determined to be positive, indicating that the implicit attitude toward one's own aging score (*D*) positively predicts the implicit self-esteem score; that is, the implicit attitude toward one's own aging negatively predicts implicit self-esteem. This indicates that the more negative the implicit attitude toward one's own aging is, the higher the implicit self-esteem level is.

## General discussion

As mentioned above, attitude toward one's own aging and self-esteem play important roles in older adults' healthy aging process. This study investigated the consistency of implicit-explicit attitude toward one's own aging, implicit-explicit self-esteem and their relations in older adults. The results support all of the proposed hypotheses.

Regarding explicit attitude toward one's own aging, the subjects in our study generally showed significantly positive explicit attitude toward their own aging; they tended to describe themselves as younger individuals. This is consistent with the results of previous research, in which researchers described how as individuals matured, their experiences of negative emotion become less severe. Older adults focus more on positive stimuli than negative in their lives and are more likely to remember positive experiences than negative ones (Cohen, 2005).

Concerning implicit attitude toward one's own aging, in this study, no significant difference was observed between the Picture and Word IAT scores of each subject, which indicates that the Picture IAT developed in this research as well as the Word IAT can effectively measure older subjects' implicit attitudes. In addition, the results indicated that the positive explicit attitudes toward aging that the subjects exhibited were not equal to their implicit attitudes toward their own aging. No significant correlation was observed between the explicit and implicit attitudes toward their own aging; in fact, the subjects had negative and passive evaluations of their own aging. This result also provides evidence for the fact that the stereotype has implicit and explicit aspects and that individuals influenced by social desirability have socially sensitive attitudes toward prejudice and stereotypes. This is consistent with the Model of Dual Attitudes, which suggests that people make two different evaluations of the same attitude object, a conscious explicit attitude and an unconscious automatically activated implicit attitude (Wilson et al., 2000). Two main features are included in the dual attitudes: once the implicit attitude is formed, it is automatically activated without consuming mental energy and motivation when the individual is faced with the attitude object because it has become habitual and automatic (Banaji and Hardin, 1996). Because the explicit attitude has not become automatic, it requires capacity and motivation to retrieve. People report their explicit attitudes when they have sufficient cognitive capacity and motivation to override their implicit attitudes; they will report implicit attitudes when they lack capacity and motivation. In addition, this difference between implicit and explicit attitudes may be derived from the moderating effect of self-presentation. Self-presentation is a method of impression management in individuals' social interactions; it reduces the consistency of the results between implicit and explicit attitudes when people do not want others to know about their real emotions or they are emotionally unwilling to accept or recognize their own emotions (Greenwald and Banaji, 1995). Nosek and Banaji (2002) noted that self-presentation served as a primary moderator of the consistency between the explicit and implicit attitudes toward one's own aging in older adults. Therefore, when using inventories to measure explicit attitudes, older subjects are likely to exhibit positive attitudes toward their own aging by self-presentation to meet social expectations or the notion of face.

The results on explicit self-esteem show that the subjects have high self-esteem, which may be caused by subjects' generally high education levels in our sample. Several studies with Chinese and non-Chinese samples demonstrated that older adults with high education levels have high socioeconomic status, and their self-esteem tends to be enhanced because they have stronger self-efficacy (Shaw et al., 2010; Xie et al., 2011). This finding is also consistent with the life-span perspective, in which growth (gain) in every stage is emphasized. Individuals at different stages of mental and physical changes also exhibit positive factors that somehow can slow down the process of aging and have the characteristic of growth (Smith and Baltes, 1999). Older adults not only experience the process of losing but also of growing in some respects. This study also observed a gender difference in implicit self-esteem: the levels of self-esteem of older men were higher than those of older women, which is also consistent with the results of previous research (Robins et al., 2002; e.g., Orth et al., 2010).

Regarding implicit self-esteem, our results demonstrate the existence of a notable implicit self-esteem effect in older adults, which indicates that they tend to link self-concept with positive attributes. However, the self-esteem effect in this study was far lower than the self-esteem effect in young subjects with similar or different cultural backgrounds (Greenwald and Farnham, 2000; Cai, 2003a,b); moreover, the implicit self-esteem effect in this study was also lower than in samples of older subjects from other cultural backgrounds (Hummert et al., 2002). This result suggests that although the older subjects in this study have the implicit self-esteem effect, compared with their high explicit self-esteem levels, their levels of implicit self-esteem are not high then.

No significant correlation was observed between explicit and implicit self-esteem, which is consistent with the results of previous studies (e.g., Bosson et al., 2000; Greenwald and Farnham, 2000) and demonstrates that they are different types of self-evaluations and separate structures. This provides robust support for the Model of Dual Attitudes (Greenwald and Farnham, 2000).

With regard to the relation between attitude toward one's own aging and self-esteem, the results of this study show a significant positive correlation between the scores on explicit attitude toward one's own aging and explicit self-esteem and between the scores on implicit attitude toward one's own aging and implicit self-esteem. The results also indicated that explicit attitude toward one's own aging could positively predict explicit self-esteem and implicit attitude toward one's own aging could negatively predict implicit self-esteem. The more positive the explicit attitude was toward one's own aging, the higher the subjects' explicit self-esteem levels were; the more negative the implicit attitude toward one's own aging, the higher their implicit self-esteem levels were. The above findings help further our understanding of the roles of attitude toward one's own aging and self-esteem in the development of older adults. That explicit attitude toward one's own aging can positively predict explicit self-esteem is consistent with the results of previous studies (e.g., Del Villar, 2014). Previous research concluded that older adults with a positive explicit attitude toward their own aging, that is, who think old individuals remain active, vital and healthy members of society, are more likely to take better care of themselves, maintain good eating and exercising patterns and experience less illness (Levy, 1975). Therefore, these older adults have higher subjective well-being and mental health levels (Levy et al., 2002; Levy, 2003a; Coudin and Alexopoulos, 2010; Scott et al., 2011). Explicit self-esteem relates closely to subjective well-being and life satisfaction (Del Villar, 2014). Thus, the older adults with positive explicit attitudes toward their own aging may have higher explicit self-esteem because of their higher subjective well-being and life satisfaction. Additionally, stereotypes have been shown to exert significant effects on the self-esteem of individuals of different groups (Sinclair et al., 2005), the results of the current research indicate that this effect also exists in the group of older adults. Existing research has determined that older adults with negative implicit attitudes toward their own aging have higher implicit self-esteem because of contrasting themselves with and differentiating themselves from the stereotypical representation of their age group (Weiss et al., 2013). Therefore, the result of the current research that links a more negative implicit attitude toward one's own aging with higher implicit self-esteem may be because subjects concentrate more on their inner selves and internalize the recognition that people have toward them as their self-knowledge, therefore influencing their levels of implicit self-esteem.

Certain limitations of this research should be considered when designing future studies. First, 70% subjects of the sample have education levels above high school, which is high in general. Subsequent research can enlarge the sample size and increase the number of subjects with various education levels. Second, only the attitude toward one's own aging and the self-esteem of older adults were investigated; thus, future researchers may be interested in investigating the differences between subjects of different age groups with regard to the two variables. Third, we have successfully created a Picture IAT for measuring attitudes toward the aging of older adults but failed to further develop a Picture IAT to measure older adults' implicit self-esteem; more attention needs to be focused on wondering how to overcome this technical problem in the follow-up study. Last, the internal reliability for the scale of “Attitudes Toward One's Own Aging” in this study was a bit low. We adopted the Attitudes toward One's Own Aging Subscale of the Philadelphia Geriatric Center Morale Scale (Lawton, 1975), and the Chinese version for this sub-scale contained only seven items. Although previous studies used Chinese sample have used this subscale to measure older adults' explicit attitudes toward aging, and reported its test-retest reliability was 0.804 (Yao et al., 1995), or reported Cronbach's α of the whole scale was 0.82 (Jiang et al., 2018). However, the number of scale items and the sample size might contribute to the low Cronbach's α value in the current study. Therefore, in the subsequent studies, researchers can consider increasing items for this scale, and enlarge the sample size, or use other standardized tools with more items.

## Conclusion

The explicit and implicit attitude toward one's own aging and explicit and implicit self-esteem are relatively independent structures. Culturally, older Chinese adults generally show more positive attitudes toward their own aging and negative implicit attitudes toward their own aging while showing higher explicit self-esteem and relatively low implicit self-esteem; their scores on explicit attitudes toward their own aging are positively correlated with their scores on explicit self-esteem. Their scores on implicit attitudes toward their own aging are also positively correlated with their scores on implicit self-esteem. Subjects who have more positive explicit attitudes toward their own aging have higher explicit self-esteem levels; and the more negative implicit attitudes toward their own aging they have, the higher their implicit self-esteem levels are. Overall, the current findings suggest that the explicit and implicit attitudes toward one's own aging and explicit and implicit self-esteem are relatively independent structures, and people's explicit and implicit attitudes toward their own aging have different predictive effects on their explicit and implicit self-esteem in different directions.

Aging generates both physiological and mental changes in older adults, including a sense of stagnation and despair. The attitudes toward aging and self-esteem of older adults are fundamental factors which determine older adults' will to live and their decisions on accepting medical aids. Thus, focusing on older adults' inner worlds, helping them form a positive attitude toward their own aging and enhancing their self-esteem are ways in which care providers can improve the quality of care and the quality of life for older adults.

## Author contributions

JC is the first and corresponding author, in charge of designing the research protocol, writing article, and dealing with the data. KZ is the second author, in charge of writing article and dealing with the data. WX is the third author, focusing on collecting data, helping record and deal with the data. QW is the fourth author, helping translate the article and join the discussion for results. ZL is the fifth author, helping correct the design and dealing with the data. YZ is the sixth author, helping correct the design and collect data.

### Conflict of interest statement

The authors declare that the research was conducted in the absence of any commercial or financial relationships that could be construed as a potential conflict of interest.
