# Supplementary material for: Does Inside Equal Outside? Relations Between Older Adults' Implicit and Explicit Aging Attitudes and Self-Esteem
Source: Front Psychol. 2018 Nov 22;9:2313. doi: 10.3389/fpsyg.2018.02313 (PMC6280639; doi:10.3389/fpsyg.2018.02313)
Supplement: Supplementary file 1 [file Presentation_1.pdf]

Appendix

Philadelphia Geriatric Center Morale Scale (PGC)

(Factor 2- attitudes toward own aging)

The Chinese version

1. 随着我年纪变老事情越来越糟。（否）
2. 我的精力和去年一样旺盛。（是）
3. 今年有更多的琐事困扰我（否）
4. 人老了自然就不中用了（否）
5. 我老了之后发现事情比原先想象得好。（是）
6. 我有时感到生命没有价值。（否）
7. 我现在和年轻时一样幸福。（是）

Self-esteem Inventory (SEI)

The Chinese version

请以下列方式标记每一个叙述：如果叙述与你通常的感觉一样，打勾表示"像我"；如果叙述与你通常的感觉不同，打叉表示"不像我"。回答无所谓对错。

1. 我花很多时间做白日梦。
2. 我对自己很有把握。

* 3. 我常希望自己是其他什么人。

4. 我容易喜欢上什么。
5. 我和我的家人在一起开心事很多。
6. 我从不为任何事担心。

* 7. 我发现当着一个团体的面讲话很难。

* 8. 我希望自己年龄再小一些。

* 9. 如果可能的话，我要改变自己的很多事。

10. 我无需太大困难就能拿定主意。
11. 我有许多开心事。

* 12. 我在家很容易不高兴。

13. 我总是做我该做的事。
14. 我对自己的工作感到自豪。

* 15. 我总是需要有人告诉我该做什么。

* 16. 我要花很长时间才能习惯新事物。
* 17. 我常对我做的事感到后悔。
18. 在与我同龄的人中我很受欢迎。
19. 我的家人通常能顾及到我的心情。
20. 我从来不会高兴。
21. 我正在做我所能做的最好的事。
* 22. 我很容易屈服。
23. 我一般能照顾好自己。
24. 我很快乐。
25. 我更愿与比我年轻的人交往。
* 26. 我的家人对我的期望太多了。
27. 我喜欢我认识的每个人。
28. 我喜欢在群体里被别人请求。
29. 我了解我自己。
* 30. 我真是太不走运了。
31. 在我的生活中，所有事情都乱成一团。
32. 人们通常会听从我的主意。
* 33. 在家里，没有人特别留意我。
34. 我从未被责骂。
* 35. 在工作上我表现得不如我希望的那么好。
36. 我能拿定主意并坚持它。
* 37. 我真不愿意做个男人 / 女人。
* 38. 我对自己评价较低。
* 39. 我不喜欢与其他人在一起。
* 40. 有许多次我想离开家。
* 41. 我从不害羞。
* 42. 我常感到不开心。
* 43. 我常为自己感到羞愧。
* 44. 我长得不如大多数人好看。
45. 如果我有事说，通常我会讲出来。

* 46. 人们经常捉弄我。

47. 我的父母了解我。

48. 我总是讲真话。

* 49. 我的老板或主管使我觉得自己不够好。

* 50. 我对自己碰到什么事并不在乎。

* 51. 我是一个失败者。

* 52. 当我被责骂时，很容易变得激动不安。

* 53. 大多数人比我更招人喜欢。

* 54. 我常常觉得我的家人似乎正在惩罚我。

55. 我总知道要对人们说些什么。

* 56. 我常常碰到令人灰心的事。

57. 通常没有什么事情能烦我。

* 58. 人们无法指责我.

说明：*为反向记分条目。
